# Novel Expandable Epoxy Beads and Epoxy Particle Foam

**DOI:** 10.3390/ma15124205

**Published:** 2022-06-14

**Authors:** Du Ngoc Uy Lan, Christian Brütting, Christian Bethke, Johannes Meuchelböck, Tobias Standau, Volker Altstädt, Holger Ruckdäschel

**Affiliations:** Department of Polymer Engineering, Universität Bayreuth, 95447 Bayreuth, Germany; christian.bruetting@uni-bayreuth.de (C.B.); christian.bethke@uni-bayreuth.de (C.B.); johannes.meuchelboeck@uni-bayreuth.de (J.M.); tobias.standau@uni-bayreuth.de (T.S.); volker.altstaedt@uni-bayreuth.de (V.A.)

**Keywords:** expandable epoxy beads, epoxy particle foam, solid-state carbamate foaming

## Abstract

Expanded polymeric beads offer the advantage of being able to produce parts with complex geometries through a consolidation process. However, established polymeric beads are made of thermoplastics, deform and melt beyond their temperature services. In this manuscript, a new technique is proposed to fabricate expandable epoxy beads (EEBs), then expand and fuse them to produce epoxy particle foams (EPFs). This technique is called solid-state carbamate foaming technique. For production of EEBs, a mixture of epoxy, carbamate and hardener is prepared and poured into a 10 mL syringe. The mixture is manually extruded into 60 °C water to obtain a cylindric shape. The extrudate is then further cured to obtain an epoxy oligomer behaving rheological tan delta 3 and 2 at 60 °C. The extrudate is cut into pellets to obtain EEBs. The EEBs are then loaded into an aluminum mold and placed in an oven at 160 °C to expand, fuse to obtain EPFs of 212 kg/m^3^ and 258 kg/m^3^. The obtained EPFs provide a T_g_ of 150–154 °C. The fusion boundaries in EPFs are well formed. Thus, the produced EPFs exhibit a compressive modulus of 50–70 MPa, with a torsion storage modulus at 30 °C of 34–56 MPa.

## 1. Introduction

In recent years, development in the field of bead foams has increased. Bead foams generally offer the possibility of producing very complex product shapes in one step, without a very expensive mold known for injection molding. Current trends include high-temperature bead foams and bead foams with special mechanical properties [1]. However, to date, research on bead foams has focused on thermoplastic bead foams, which are deformed, melted and dripped beyond their temperature services. Furthermore, high-temperature bead foams require a high processing temperature during foaming, as well as high energy consumption. There are two types of thermoplastic beads, namely expanded beads and expandable beads. Both types of beads are used to produce particle foams by a steam-chest molding process [1].

**Scheme 1:**
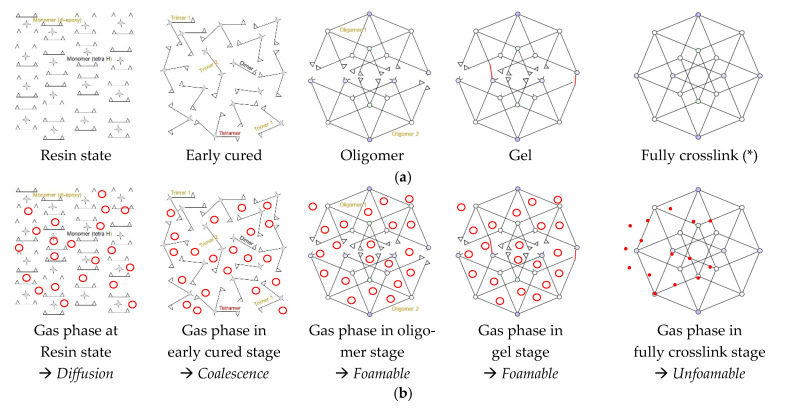
(**a**) Sample of 32 monomer units of a difunctional epoxy resin (diepoxy) and 16 monomer units of a hardener with four H-active (tetra H) (*) [6]. (**b**) Introduction of gas phase in the resin stage, early curing stage, at oligomer stage and gel state, as well as gas in the fully crosslinked state.

Thermoplastics also require a suitable range of molecular weight (M_w_), which supports the processing window for proper foaming. Chain extenders can be added to adjust the M_w_ from a low to a high value to improve foaming [2,3]. This practice not only results in a higher M_w_ but also more branching in the polymer chains. Unlike thermoplastics, epoxy resin and hardener undergo step polymerization to form a final three-dimensional crosslinked network (3D crosslink). The mixture of epoxy resin and hardener transitions different states from resin state to an early cured state (dimer, trimer and tetramer), to an oligomer state, to a gel state and, finally, to a fully crosslinked state, as shown in Scheme 1a. The initiation of a gas phase by a chemical reaction can take place at different states, as shown in Scheme 1b. However, the introduction of a gas phase in the resin state leads to high gas diffusion and foam coalescence. The introduction of gas phase in early curing results in the same issues unless a high-M_w_ epoxy and hardener are used. The introduction of the gas phase at the oligomer state supports and improves foam formation. The introduction of the gas phase at the gel state results in an expansion, depending on the molecular weight between the crosslinks (M_c_). Finally, the introduction of the gas phase in the fully crosslinked network does not produce a foamable system and leads to cracking. This research is focused on the introduction of the gas phase in the oligomer state to foam the epoxy oligomer network. Further curing is carried out to obtain a full 3D crosslinking network. This foaming technique is called solid-state foaming. This solid-state foaming or foaming of epoxy oligomer is capable of foaming epoxy with gaseous physical CO_2_ under high pressure, as shown in our previous study [4]. Recently, solid-state epoxy has been applied to develop epoxy molding compounds (EMCs) for the production of electronics parts using a single process step of injection molding technology. EMC pellets consist of plasticizers, adhesion promoters, thinners or lubricants and are used in transfer molding or injection molding [5].

**Scheme 2:**
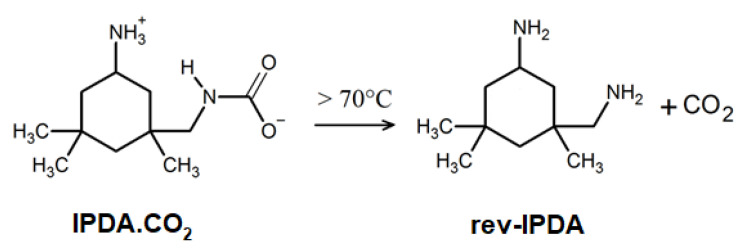
Decomposition reaction of IDPA.CO_2_ carbamate to revive the IPDA (rev-IPDA) and CO_2_ gas [8].

The use of carbamate is beneficial for this solid-state method [7,8,9]. As a product of amine and carbon dioxide, carbamate decomposes at elevated temperatures to release CO_2_ gas and recovered amine. Scheme 2 shows the decomposition of IPDA.CO_2_ carbamate with a decomposition temperature above 70 °C. As a first advantage of this system, oligomers with different moduli can be developed from the reaction of epoxies and pure-amines. The development of oligomers is achieved not only by different precuring levels of an epoxy-hardener system but also by different types of epoxy resins and hardeners. The aim of this work is to develop oligomers that undergo the transition to solid state at room temperature. In fact, these solid-state epoxies are carbamate-filled epoxy oligomers. They can be easily shaped into pellets or flat sheets [10]. These expandable epoxy preforms are suitable for designing epoxy foam products with various geometries. In addition, epoxy products can be produced with the desired density. The second advantage of the carbamate-epoxy system is the control of the cellular structure. The newly formed cellular structure is stabilized by the reaction of epoxy-oligomer and the revived amine. This reaction results in a larger network and higher modulus of the matrix, thus strengthening the cellular structure. Therefore, foaming epoxy with carbamates provide better control than foaming with other chemical blowing agents. The third advantage of carbamate is the use of CO_2_ as a blowing agent, which is less toxic compared to other blowing agents, such as toluene-4-sulfonohydrazide (TSH) [11] and azodicarbonamide (ADC) [12]. Solid-state carbamate foaming allows for a larger product shaping process compared to direct foaming of resins. Currently, all commercially epoxy foams are available as two- or three-component resins and are made directly from resins such as Sicomin, Resotech, Biresin EA200, Ampreg-F2301 and Neukadur EP RF [13,14,15,16,17].

Besides the use of chemical and physical blowing agents to foam epoxy, porous beads are also added directly to resin and have worked as a void template for production of syntactic foams. Research on porous beads has recently gained attention due to their excellent thermal properties. Porous microbead provide a large surface area and are used as an additive for the production of syntactic thermoset foams [18,19]. The most common techniques for fabricating porous microbead are based on immiscibility of resin materials and a medium phase, such as epoxy in water [20] and epoxy in oil [21]. The porous beads are formed by an emulsification technique or a dropping technique [22]. Conductive material such as carbon black can be added to produce conductive epoxy porous beads [23,24]. In this technique, chemical blowing agents are used, and the foaming process is initiated in the resin state or in the early cured state. Therefore, the technique is highly dependent on the decomposition temperature of the blowing agents (such as TSH, sodium bicarbonates or ammonium bicarbonates) [22,23,24]. Fusion of these porous microbeads is not possible because they are fully cured, and an epoxy binder is needed to act as a matrix and to form the product shape.

In this work, we propose a novel technique to produce expandable epoxy beads (EEBs) by extrusion process to ensure scalability. Ancamine 2914UF, an ultra-fast curing hardener, and DEN 431 epoxy Novolac are used to form the oligomer network. Ancamine hardener is produced by Evonik and contains an ionic liquid based on polyamine and p-toluenesulfonic acid [25]. Carbamate derived from isophorone diamine (IDPA) is used as both a blowing agent and a latent curing agent. EEBs are developed based on the rheological characteristics of the epoxy oligomer, such as storage modulus, loss modulus and tan delta. As the ratio of loss modulus to storage modulus, tan delta presents the damping and viscoelastic behavior. According to our previous study, the epoxy oligomer changes from viscous-dominated to elastic-dominated behavior due to further crosslinking reactions. This affects the foaming performance. Therefore, the EEBs are precured at 60 °C to different tan delta values. Finally, their expansion and fusion ability to form EPFs are investigated.

## 2. Materials and Methods

### 2.1. Materials

Epoxy Novolak (D.E.N 431) was purchased from Olin, Germany. D.E.N 431 resin has an epoxide equivalent weight of 173.5 g/eq and a viscosity of 1100–1700 mPa·s at 25 °C. Ancamine 2914UF was provided by Evonik, Germany. Ancamine 2914UF has an equivalent weight per H active (AHEW) of 95 and a viscosity of 300–2000 mPa·s at 25 °C. Carbamate IPDA.CO_2_ was synthesized in-house as described in a previous report and has an M_w_ of 214.3 g/mol and an AHEW of 53.6 g/mol [8]. The decomposition temperature is about 70 °C, with a CO_2_ content of IPDA.CO_2_ of 21 wt%. 

### 2.2. Methods

Formula development: A blend of A2914 and carbamate IPDA.CO_2_ is prepared with an AHEW proportion of 15:85 (A2914:IPDA.CO_2_). When the weight of the epoxy resin is defined as m_epoxy_, the weight of IDPA.CO_2_ and A2914 is calculated as in Equation (1) and Equation (2), respectively. The theoretical weight percentage of CO_2_ is determined as in Equation (3). The weight of each material to obtain a mixture of 16.4 g is summarized and presented in Table 1.

(1) $$m_{\mathrm{IPDA}.\mathrm{CO}2}=85\%\times \frac{53.6}{173.5}\times m_{\mathrm{epoxy}}$$

(2) $$m_{A2914}=15\%\times \frac{95}{173.5}\times m_{\mathrm{epoxy}}$$

(3) $${\mathrm{CO}}_{2}\;\left(\mathrm{wt}\%\right)=\frac{21\%\times m_{\mathrm{IP}.\mathrm{CO}2}}{m_{\mathrm{epoxy}}+m_{\mathrm{IP}.\mathrm{CO}2}+m_{A2914}}$$

Formula compounding: D.E.N 431 and IPDA.CO_2_ carbamate were weighted into a 60 mL polypropylene (PP) cup. They were mixed using a DAC 150 speed mixer (Hauschild Engineering, Hamm, Germany) at 3000 rpm for one minute. The correct amount of 2914UF was added and also mixed at 3000 rpm for one minute. 

**Figure 1:**
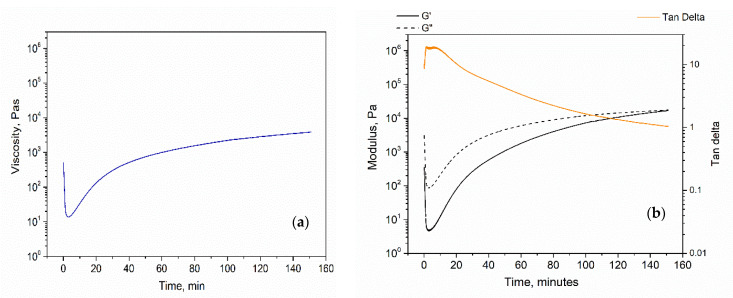
Rheological behavior EN.A.IDPA.CO_2_ precured at 60 °C for 150 min. (**a**) Viscosity behavior, (**b**) G’, G” and tan delta.

Rheological behavior of DEN431-2194-IDPA.CO_2_: The rheology of the epoxy mixture was observed using an Anton Paar MCR301 rheometer (Graz, Austria) with a 25 mm parallel plate. The sample thickness was 1 mm and was tested at ω = 1 rad/s and γ = 3%. The specimen was precured at 60 °C for 150 min. Figure 1a shows the viscosity of the EN.A.IDPA.CO_2_. The time to reach a viscosity of 180 Pa·s (20 min) is indicated. Figure 1b shows the storage modulus (G’), loss modulus (G”) and tan delta versus precuring time. The precuring time on the x-axis to obtain tan delta 3 and tan delta 2 on the right y-axis was identified as 64.5 min and 86.5 min, respectively. The rheology of thermoset depends, to a considerable extent, on the oscillatory strain, γ. The monomers transfer into dimers, trimers, oligomers, gels and completely crosslink. The storage modulus increases significantly during these phases. Therefore, a higher oscillatory strain, γ, leads to a lower storage modulus and a different tan delta. In this study, an oscillatory strain, γ, of 3% was chosen, which is suitable to observe the mechanisms of precuring, complete curing and foaming.

Expandable epoxy beads from extrusion process: The epoxy mixture was transferred into two 10 mL PP syringes and cured in a conventional oven at 60 °C. The mixture was precured to a viscosity of 180 Pa·s. A viscosity of 180 Pa·s was selected to ensure that the extrudate formed a cylindric shape during further precuring at 60 °C. The epoxy mixture was extruded into a polypropylene tray containing deionized water at a temperature of 60 °C. The epoxy extrudate in water was continued to cure to a tan delta value of 3 (to 64.5 min) and tan delta of 2 (to 86.5 min) in a conventional oven. Next, the water was poured out in a beaker, and the extrudate was cooled down to room temperature (25 °C). They were then cut into 20 cm length and stored at 0 °C in a fridge (for 24 h). 

Free expansion in oven: Ten pellets were used to undergo a free expansion at 140 °C, 150 °C and 160 °C in a conventional oven for 60 min at each temperature. The difference in weight before and after free expansion was determined and compared with the theoretical CO_2_ percentage (Table 1). 

**Figure 2:**
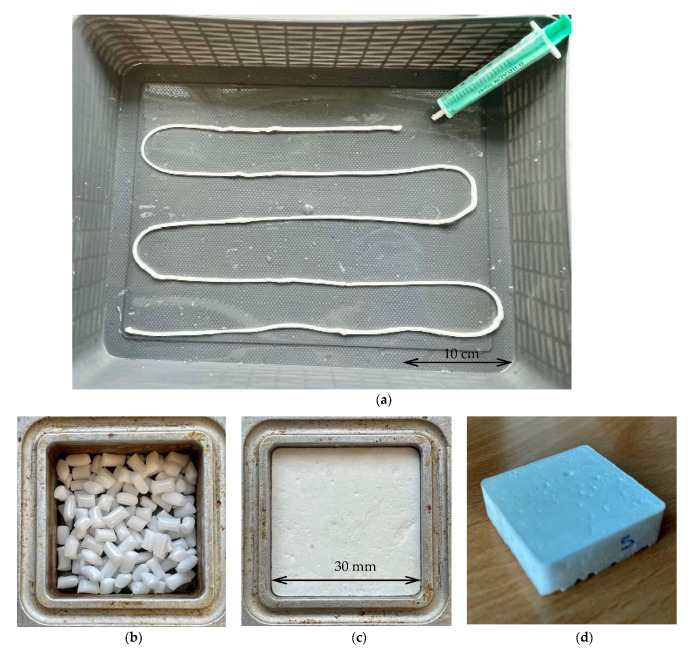
(**a**) Expandable epoxy extrudate with a diameter of 2.7–3.5 mm and a length of 1.5 m, (**b**) EEBs in mold, (**c**) EPF after foaming at 160 °C and cooling and (**d**) EPF after demolding.

Thermal foaming fusion in a closed mold: An aluminum mold manufactured in-house with a 30 mm × 30 mm × 10 mm inner rectangular cuboid (volume of 9 cm^3^) was used. The extrudate was weighted to the target densities of 210 kg/m^3^ (1.93 g) and 258 kg/m^3^ (2.35 g). They were cut into pellets and placed into the aluminum mold. The mold was closed with four screws. Expansion was conducted in a conventional oven to ensure a consistent foaming temperature of the surrounding environment. The details of the thermal foaming and fusion are shown in Table 2. Cooling was conducted for 2 min with two aluminum plates (20 cm × 20 cm × 2 cm). The epoxy particle foam was unmolded for further testing. Figure 2 shows the expandable epoxy extrudate and the thermal fusion process of EEBs.

### 2.3. Characterization and Testing

Rheological behavior was observed using an Anton Paar MCR301 rheometer (Graz, Austria) equipped with a 25 mm parallel plate. The sample thickness was 1 mm and tested with ω = 1 rad/s and γ = 3%. The rheological behavior was investigated to simulate solid-state processing: 1st: heating from 30 °C to 60 °C in one minute; 2nd: precuring at 60 °C to tan delta 3 and 2; 3rd: cooling down from 60 °C to 30 °C (a rate of 10 K/min) and hold at 30 °C for 30 s; 4th: heating up from 30 °C to 160 °C with 30 K/min to allow foaming; and 5th: foaming stabilization and full curing at 160 °C for 60 min. 

The T_g_ and curing temperature of EEBs were studied by differential scanning calorimetry using a Mettler Toledo DSC1 Star system (Gießen, Germany). Scanning was conducted from −20 °C to 200 °C in a nitrogen (N_2_) environment with a flowrate of 50 mL/min and a heating rate of 10 K/min. T_g_ and T_onset_ of epoxy oligomer and revIPDA were reported.

The densities of the EEBs, epoxy bead foam and EPFs were measured according to Archimedes’ principle using an AG245 analytic balance from Mettler Toledo (Gießen, Germany) equipped with a density kit. EPF with dimensions of 30 mm × 30 mm × 10 mm was used. 

The surface morphology and the cross surfaces of the epoxy particle foams were observed using a 3D profilometer (Keyence, Neu-Isenburg, Germany). The cellular morphology of the EPF was observed using an SEM-Jeol JSM-6510 instrument (Tokyo, Japan). The samples were sputtered with a 13 nm thick gold layer using a Cressington 108auto sputter coater (Watford, England, UK) to eliminate the charging effect during SEM observation.

Mechanical compressive test according to DIN ISO 844: Epoxy particle foams with dimensions of 30 mm × 30 mm × 10 mm were used. The test was conducted at a rate of 1 mm/min to 75% strain. A compressive test was conducted using a Z050 universal test machine (Zwick GmBh, Ennepetal, Germany) with a load cell of 50 kN. An average of three measurements was obtained and discussed. The energy absorption efficiency (η) was calculated by Equation (4) [26,27]. The densification strain was adopted from the maximum value of the energy absorption efficiency.

(4) $$\eta_{\left(\varepsilon \right)}=\frac{\int_{0}^{\varepsilon }\sigma \left(\varepsilon \right)d\varepsilon }{\sigma \left(\varepsilon \right)}$$

$\eta_{\left(\varepsilon \right)}$: energy absorption efficiency
σ(ε): compressive stress 
ε: compressive strain.

Torsional dynamic mechanical analysis: The storage modulus and glass transition temperature of the epoxy foams were measured using dynamic mechanical analysis (DMA). The test was performed with a sample size of 50 mm × 10 mm × 3 mm and 50 mm × 10 mm × 4 mm using an RDA III instrument (TA Instruments, New Castle, DE, USA). The specimens were heated from 25 to 220 °C at a heating rate of 2 K/min and measured at a frequency of 1 Hz.

## 3. Results and Discussion

### 3.1. Rheological Behavior of DEN431-2914-IPDA.CO_2_

**Figure 3:**
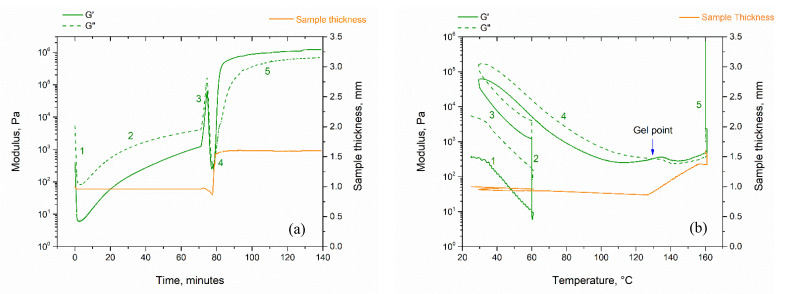
Rheological behavior of DEN431-2914-IPDA.CO_2_ showing storage modulus (G’), loss modulus (G”) and sample thickness: (**a**) versus time, (**b**) versus temperature. 1st: heating from 30 °C to 60 °C; 2nd: curing at 60 °C to tan delta 3; 3rd: cooling down to 30 °C and hold for 30 s; 4th: heating up from 30 °C to 160 °C; and 5th: foam stabilization and curing at 160 °C.

**Figure 4:**
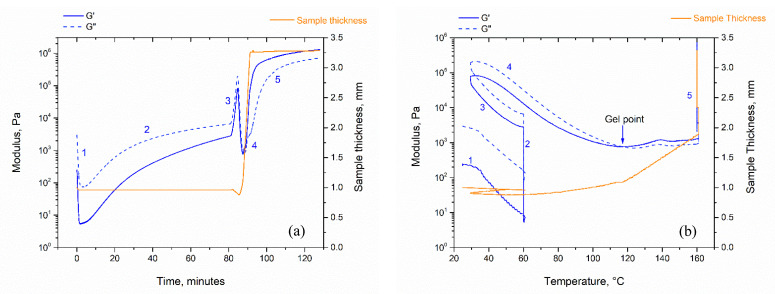
Rheological behavior of DEN431-2914-IPDA.CO_2_ showing storage modulus (G’), loss modulus (G”) and sample thickness: (**a**) versus time, (**b**) versus temperature. 1st: heating from 30 °C to 60 °C; 2nd: curing at 60 °C to tan delta 2; 3rd: cooling down to 30 °C and hold for 30 s; 4th: heating up from 30 °C to 160 °C; and 5th: foam stabilization and curing at 160 °C.

To study the curing reaction between Den 431 epoxy and 2914UP curing gent, the solid-state behavior at 30 °C and the rheological behavior during the foaming process of carbamate, as well as the further curing between the revived IDPA and epoxy oligomers, the time–temperature profile during these processes was simulated in a plate rheometer. Figure 3 and Figure 4 show the storage modulus (G’) and the loss modulus (G”) on the left y-axis and the sample thickness on the right y-axis, both versus time and temperature. Because precuring was conducted to achieve tan delta 3 and tan delta 2 at 60 °C, gelation was not yet achieved. When cooled down to 30 °C, the carbamate-filled epoxy oligomers (CfEO) tan 3 and tan 2 had a G’_30°C_ of 30 kPa and 48 kPa, respectively. These values indicate the solid-state behavior of EEB−tan 3 and EEB−tan 2, respectively. When heated from 30 °C to 160 °C, the CfEO was foamed, and the sample thickness obviously increased (as shown in Figure 3a and Figure 4a). During this heating process, gelation also occurred, as shown by the intersection of G’ and G”. It is interesting to note that the thickness of 1 mm increases significantly around the gel point, as shown in Figure 3b and Figure 4b. The gelation network is strong enough to expand vertically. Furthermore, the growth in sample thickness of CfEO tan−2 started at a very early temperature of 70 °C in the same range as the decomposition temperature of IPDA.CO_2_ to release CO_2_ [8]. The sample thickness increased significantly at 117 °C. The G’_foaming_ of CfEO tan−2 was 0.74 kPa (Table 3), indicating that CfEO tan−2 had a high M_w_, which was sufficient to hold and expand with the CO_2_ released from carbamate. Besides rheological measurements, thermomechanical analysis (TMA) was also used to study the expansion thickness of a microsphere-filled epoxy, as reported in [28]. In order to obtain more foaming characteristics, such as foam modulus and foam tan delta, a rheological measurement is more appropriate. 

**Figure 5:**
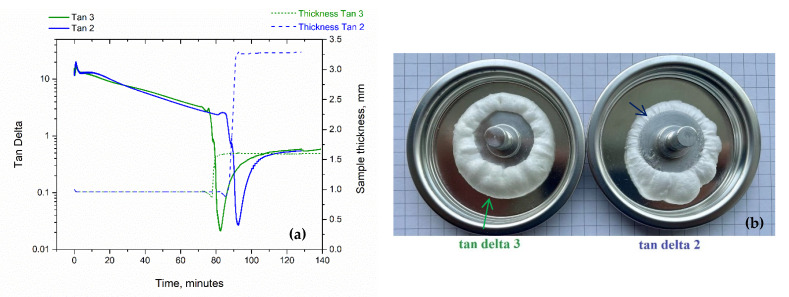
(**a**) Tan delta and sample thickness changing from solid state to foam versus time. (**b**) Rheological foam sample of tan 3 showing a soft texture, whereas tan 2 shows a rougher texture.

Figure 5a shows the evolution of tan delta from the resin state to oligomer to the foaming and fully cured states. The sample thickness (right y-axis) of CfEO tan−2 was higher than that of CfEO tan−3, but both foamed and expanded in different patterns. As shown in Figure 5b, the rheologic foam sample of CfEO tan−3 foamed and flowed simultaneously and obtained a smoother foam surface. In an obviously different pattern, the rheologic foam sample of CfEO tan−2 expanded more upwardly and obtained a rougher foam surface. These foam patterns were fully supported by the G’_foaming_ and G’_gel_ of CfEO tan−3 and CfEO tan−2, as shown in Table 3. Therefore, two EEBs with different foaming behaviors were prepared by the solid-state carbamate foaming technique. This is an advantage of this solid-state foaming method compared to the resin-direct foaming method. The solid-state foaming technique affords better control over foaming and expansion than the resin-direct foaming method. 

### 3.2. EEB Properties and Density of Epoxy Beads

**Figure 6:**
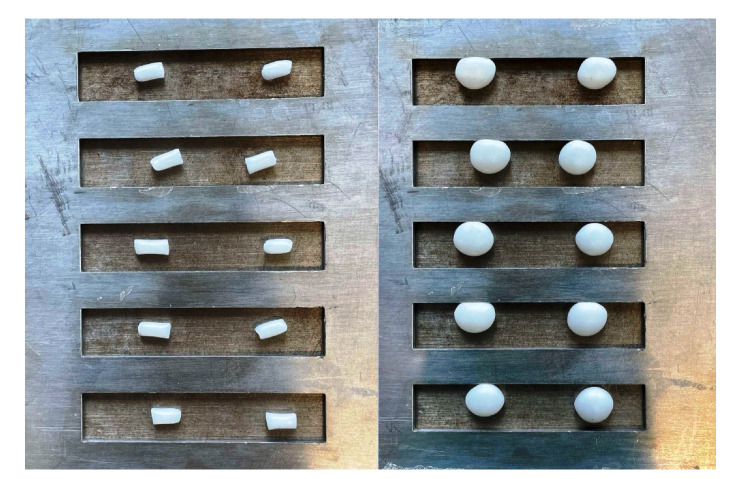
Tan−3 EEBs (**left**) and their expanded epoxy beads after foaming at 150 °C (**right**).

The glass transition of EEB−tan 3 and EEB−tan 2 was in a range of 8.5–9.5 °C, as measured by DSC. The further curing temperature for both EEBs was 105–106 °C based on the DSC result. Therefore, they behave like a solid at room temperature, as shown in Figure 2a,b and Figure 6a. These EEBs are novel, expandable beads that are well-suited for shaping products with complex geometry. The EEBs have a solid density of 1.05–1.09 g/cm^3^. 

Figure 6 shows ten tan−3 EEBs and their expanded epoxy beads after foaming at 150 °C in the oven. The expanded epoxy beads were spherical and white. After foaming in the oven, the weight loss of EEB−tan 3 and EEB−tan 2 was quite similar (4.5–4.7%) and higher than the theoretical CO_2_ content of 4.1%, as shown in Table 4. There is a possibility that some revived IPDA was evaporated during foaming. As expected, foaming at a higher temperature results in a lower density, which means that more expansion is achieved. The expanded epoxy beads of EEB−tan 3 also have a slightly lower density than expanded epoxy beads from EEB−tan 2. It is important to note that only DEN431 and 2914 reacted during precuring at 60 °C. The stoichiometric ratio of 2914 is 15%. Thus, the epoxy oligomer networks of tan delta 3 and tan delta 2 after precuring at 60°C are not significantly different. This difference has a small effect on the free expansion of EEBs. The lowest density of epoxy beads was about 130–132 kg/m^3^ at a foaming temperature of 160 °C. Therefore, the foaming temperature of 160 °C was selected to produce EPF with a target density of 210 kg/m^3^. The temperature of 160 °C is also slightly above the T_g_ (146.0–148.0 °C) of the fully cured epoxy system based on DSC results. 

### 3.3. Morphology of EPF after Foaming and Curing at 160 °C

The weight loss after foaming is quite similar to the weight loss of EEBs after free expansion in the oven. An amount of 1.92–1.94 g was used to achieve a target density of 210–214 kg/m^3^ compared to 2.35 g for a density of 258 kg/m^3^. Thus, there was more free volume in the mold, and the weight loss was slightly higher for a density of 210–214 kg/m^3^.

**Figure 7:**
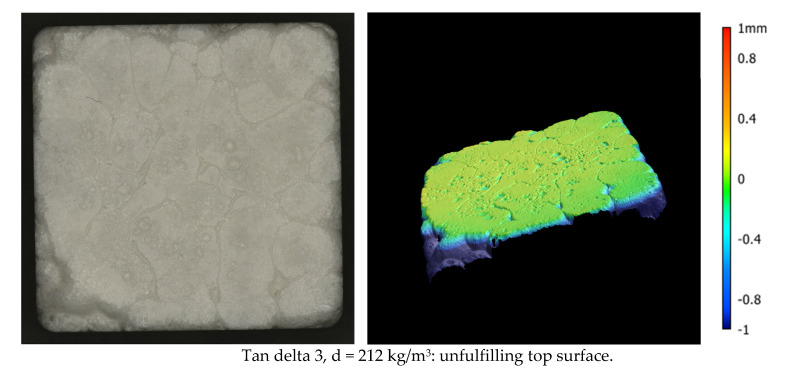

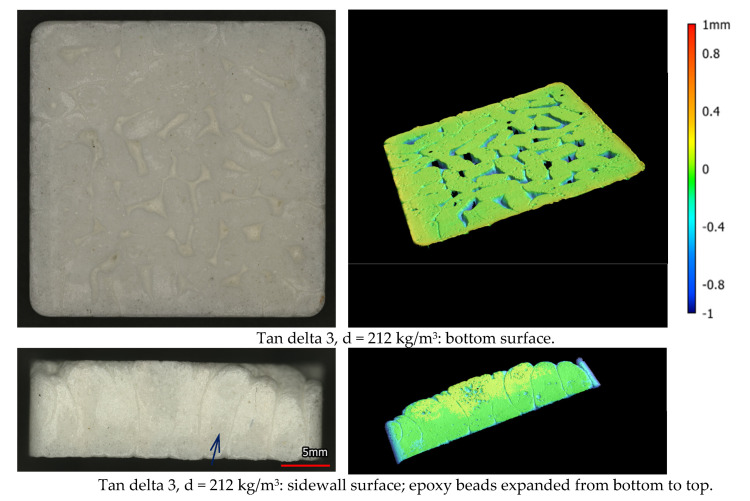
Surface morphology of epoxy particle foam from EEBs with tan delta 3; the unfulfilling top surface indicates that 215 kg/m^3^ is the lowest attainable density.

**Figure 8:**
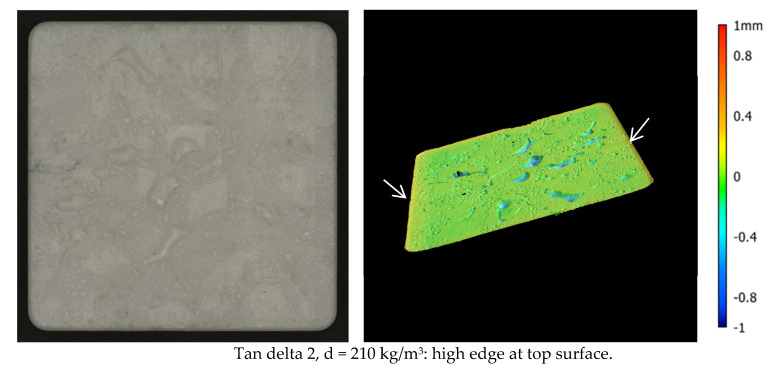

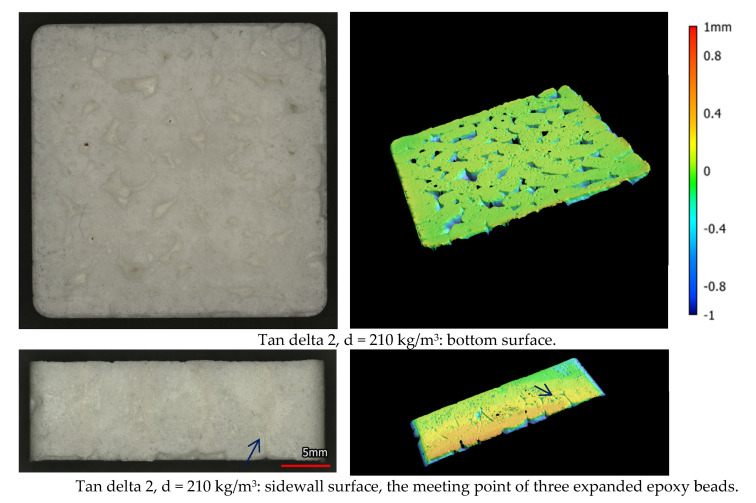
Surface morphology of epoxy particle foam from EEBs with tan delta 2; achieved lower density (210 kg/m^3^) compared to EEB−tan delta 3.

**Figure 9:**
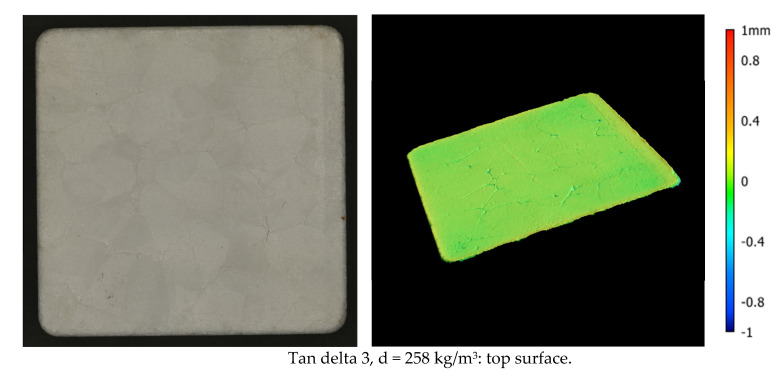

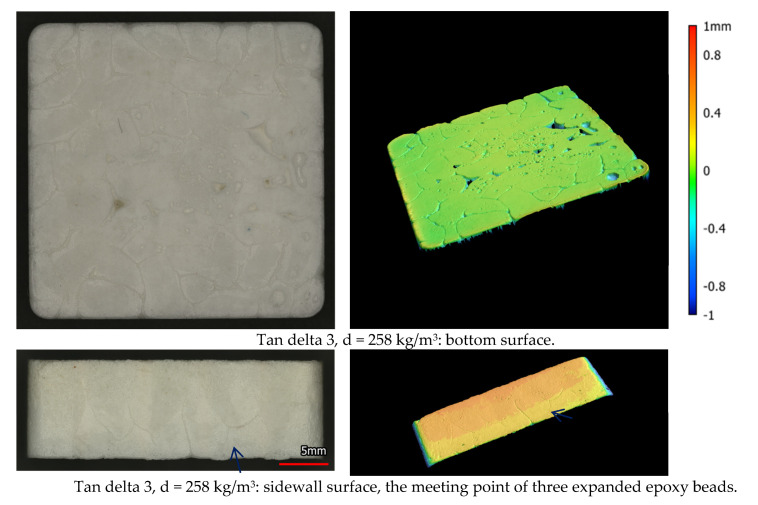
An improved surface morphology of epoxy particle foam from EEBs with tan delta 3 with a density of 258 kg/m^3^.

**Figure 10:**
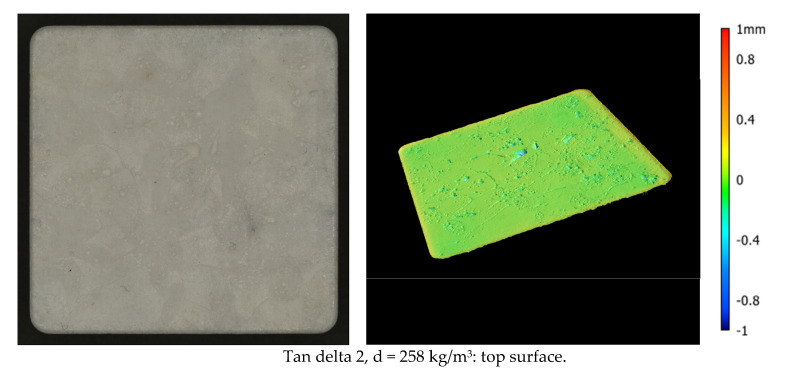

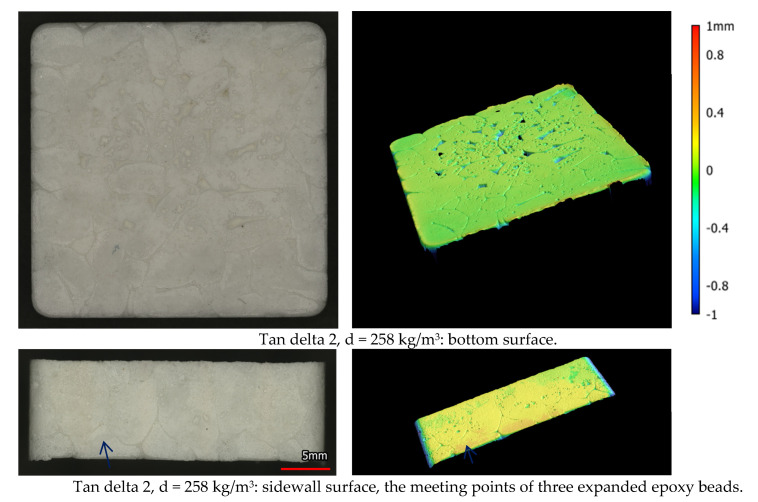
Improved surface morphology of Epoxy particle foam from EEBs with tan delta 2 with a density of 258 kg/m^3^.

EPFs with two densities were prepared, and their top, bottom and cross surface at the center EPF were observed using a 3D profilometer (Keyence, Germany), as shown in Figure 7, Figure 8, Figure 9 and Figure 10. The EPF of EEB−tan 3 was not able to obtain a fulfilling top surface at a density of 215 kg/m^3^, as shown in Figure 7. An amount of 1.94 g of EEB−tan 3 was used to produce EPF with a density of 215 kg/m^3^, which is higher than the amount of EEB−tan 2, i.e., 1.92 g. A fusion border was observed on the top and the bottom surface. However, the bottom surface showed obvious fusion gaps between the EEB. EEBs at the bottom of the mold received better heat transfer and expanded at a very early state. Due to the size of EEBs (2.7–3.5 mm in diameter and 3.0–4.0 in length) they were too large for the 30 mm × 30 mm × 10 mm mold (shown in Figure 2b). The sidewall surface shows that an EEB was able to expand and rise to the top mold.

The EPF using EEB−tan 2 was able to achieve a lower density of 210 kg/m^3^. The surface morphologies are shown in Figure 8. The high edge on the top surface proves the different foaming behavior of EEB−tan 2 compared to EEB−tan 3. This result is in agreement with the rheological behavior shown in Figure 3, Figure 4 and Figure 5. Moreover, the fusion border is less pronounced on the top surface. However, the bottom surface shows more obvious fusion gaps because EEB−tan 2 tends to expand upward. The sidewall shows meeting points of three expanded epoxy beads.

The EPF using EEB−tan 3 with a density of 258 kg/m^3^ has a smooth top surface, as seen in Figure 9. The fusion border is less characteristic on the top surface. The bottom surface is also improved and has fewer fusion gaps between the EEBs. There is also no fusion border on the cross section. The cell size of the EPF using EEB−tan 3 at 258 kg/m^3^ was much smaller than that at 215 kg/m^3^ (Figure 7). This proves that EPFs with various densities can be developed from these novel EEBs. The sidewall shows the meeting points of three expanded epoxy beads.

As expected, EPF using EEB−tan 2 with a density of 258 kg/m^3^ also has an improved top surface, as shown in Figure 10. The top surface has some bubbles and fewer fusion borders in EPF using EEB−tan 2 compared to EPF using EEB−tan 3 at a similar density. The bottom surface is also improved and has fewer fusion gaps among the EEBs. There is no fusion border on the cross section. In general, both EEB−tan 3 and EEB−tan 2 exhibit good foaming and fusion to produce EPF with the lowest density of 210 kg/m^3^. The different foaming temperature could affect the morphology of the bottom surface, and a larger mold can be used to improve this effect. The meeting points of three expanded epoxy beads are also shown on the sidewall surface.

### 3.4. Morphology of Cellular Structure by SEM 

In order to achieve an improved understanding, some important information about the sample and processing features should be noted. First, the EEBs were lying on the bottom of the mold with free space on top, as shown Figure 2b. Second, the EEBs foam, flow and expand from the bottom and rise to fill the top space. Third, an EEB (density of 1056–1090 kg/m^3^) with a diameter of 2.7–3.5 mm and a length of 3.0–4.5 mm would expand by more than five times to a density of 210–212 kg/m^3^ and more than four times to a density of 258 kg/m^3^, respectively. Fourth, the SEM surface dimensions of 10 mm in height and 4 mm in width could belong to one, two or three expanded epoxy beads. Finally, three sections images were captured via SEM to assemble the top, middle and bottom surfaces. The assembly height of the four EPFs was not similar because the cellular morphology of the top, middle and bottom surfaces SEM overlapped differently. 

**Figure 11:**
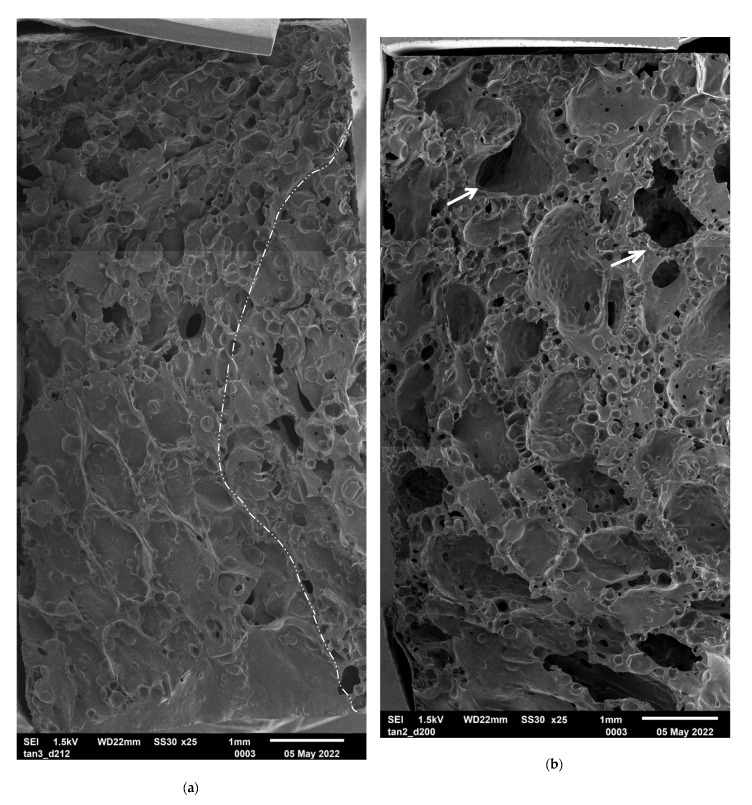
Assembled SEM of (**a**) EPF 212 kg/m^3^ made of EEB−tan 3, the dash-dot-dot line shows a fusion boundary and (**b**) EPF 210 kg/m^3^ made of EEB−tan 2.

EEB−tan 3 and EEB−tan 2 have completely different morphologies at the two target densities. The foaming behavior of the rheological sample (Figure 5b) shows that EEB−tan 3 foams and flows more horizontally, whereas EEB−tan 2 expands more vertically. As expected, EPF 212 kg/m^3^ made of EEB−tan 3 does not show a sharp cellular structure with clear cell walls from the lower to the middle and to the upper EPF, as seen in Figure 11a. EEB−tan 3 foamed at the bottom and rose to the top, resulting in more large cells on the upper EPF. EPF from EEB−tan 2 with a density of 210 kg/m^3^ exhibited a completely different cellular structure with a sharp cellular structure and a clear cell wall, as shown in Figure 11b. It is important to note that the large, irregular voids in the upper EPF made of EEB−tan 2 are not coalescent cells (indicated by the arrows). They originated as free volume on the top of the EEBs within the mold. EEB−tan 2 expanded and encapsulated them into the cellular structure. In addition, the gelation of EEB−tan 2 occurred earlier, so the cellular structure was also stabilized faster.

**Figure 12:**
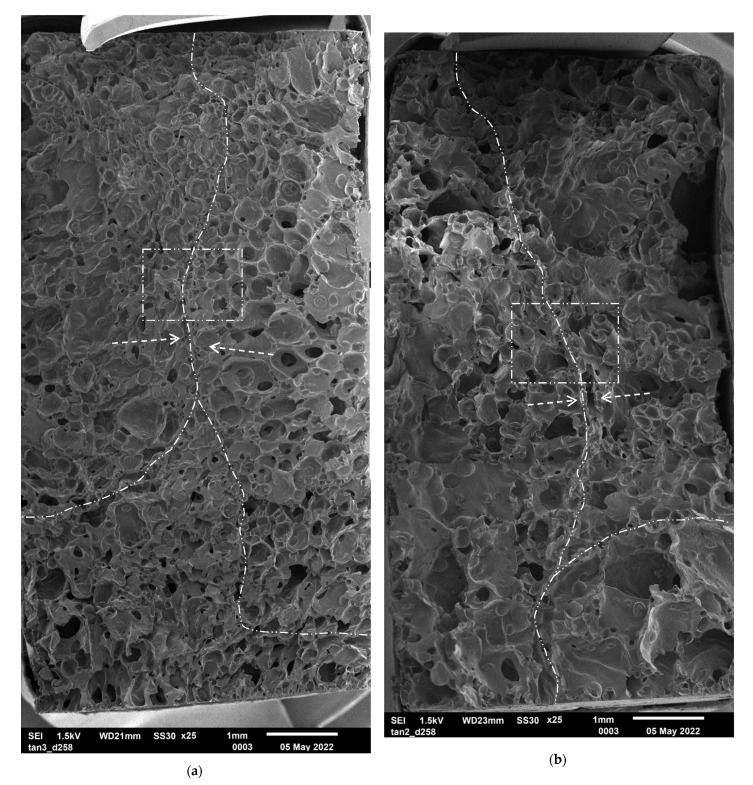
Assembled SEM of EPFs with a density of 258 kg/m^3^ showing the fusion boundary, with arrows indicating the compressed cellular in vertical direction (**a**) made of EEB−tan 3 and (**b**) EEB−tan 2. A zoomed-in image with the dash-dot-dot rectangular is shown in Figure 13.

As expected, a fusion boundary is observed in the EPFs with a density of 258 kg/m^3^ made of EEB−tan 3 and EEB−tan 2, as shown as the dash-dot-dot lines in Figure 12a,b respectively. The fusion boundary was clearly identified as the region exhibiting small cells from both expanded epoxy beads. This is totally different from the welding border of pre-expanded thermoplastic beads, such as polystyrene or polycarbonate [29]. The cellular morphology in Figure 12a shows the flow and fusion of the expanded EEB−tan 3 from two visible directions (indicated by the arrow). In a dissimilar pattern, two tan−2 EEBs restricted their expansion and formed a fusion region consisting of compressed cells (indicated by the arrow). These compressed cells are in the rising direction, as seen in Figure 12b.

**Figure 13:**
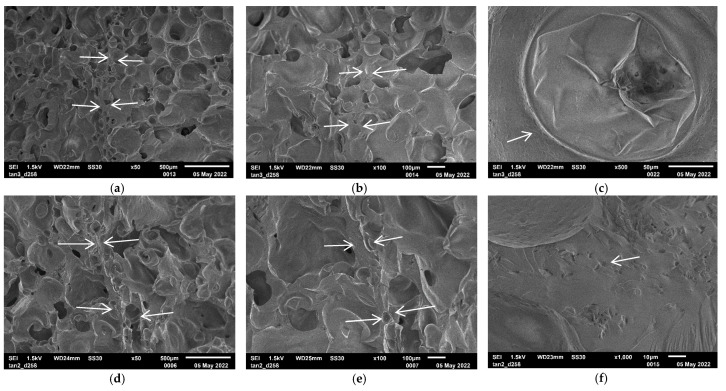
Assembled SEM of EPFs with a density of 258 kg/m^3^ made of EEB−tan 3: (**a**) showing the fusion boundary indicated by the arrows, (**b**) firm boundary of EPF-EEB−tan 3 (**c**) microcell on the foam skeleton; and EPFs made of EEB−tan 2: (**d**) showing the fusion boundary, arrows indicate the compressed cells in the vertical direction, (**e**) thicker boundary, (**f**) microcell on the boundary.

The fusion boundary of EPF made of EEB−tan 3 is shown in Figure 13a,b, and that of EEB−tan 3 is shown in Figure 13d,e (indicated by the arrow). The boundary is consolidated and does not show the boundary border between two expanded epoxy beads. As expected, EEB−tan 3 formed a thinner fusion boundary (Figure 13b), whereas EEB−tan 2 formed a thicker fusion boundary (Figure 13d). In addition, the fusion boundary and the cell wall, as well as the cell surface, consist of microcells, as seen in Figure 13e,f. Solid-sate carbamate foaming results in a highly porous structure.

**Figure 14:**
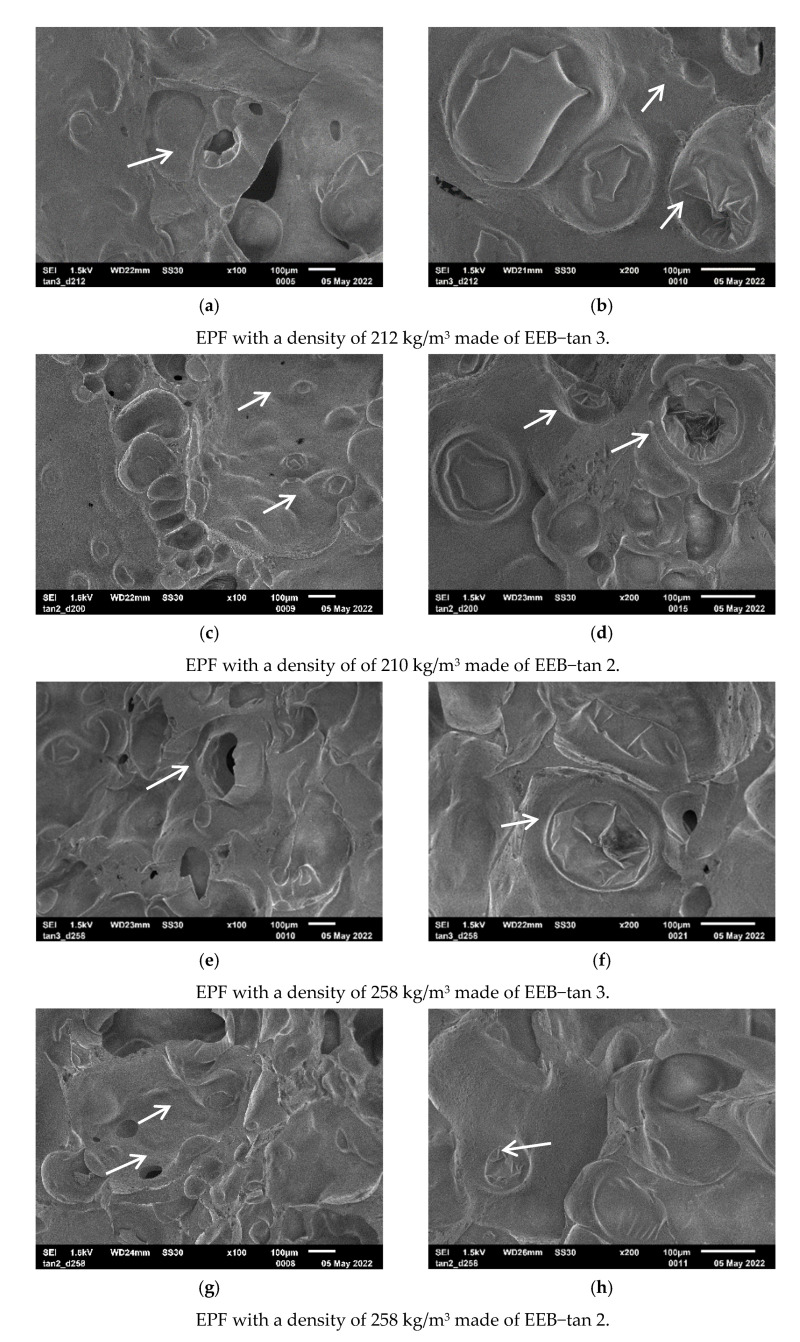
Cell morphologies of EPF made of EEBs with different densities. (**a**) Blooming cell, (**b**) wrinkled cell and dented cells, (**c**) dented cells, (**d**) wrinkled cells, (**e**) blooming cell, (**f**) wrinkled cells, (**g**) dented cell and blown cell, (**h**) wrinkled cell.

Figure 14 shows a unique expansion feature of EEB−tan 3 and EEB−tan 2. The cells were inflated and expanded by the CO_2_ from the carbamate. Some cells in the EPFs made of EEB−tan 3 were stretched and bloomed, showing thin walls as indicated by the arrow in Figure 14a,e. Many cells were expanded and dented, as indicated by the arrow and shown in Figure 14b,c and g. No cell rupture was observed in EPF made of EEB−tan 2 due to the higher storage modulus. On the other hand, some stretched cells shrank and caused many wrinkles, as indicated by the arrow and shown in Figure 14b,d,f,h. This indicates that the cell wall was stretched very thin and behaved more elastically. This is consistent with rheological results. More wrinkled cells formed in the EPF with a density of 210 kg/m^3^ made of EEB−tan 2 (Figure 14d) because there was more free space in the mold for expansion. The cellular structure proves the advantage of solid-state carbamate foaming. The revived IPDA continued to react with the epoxy oligomer and strengthened the cell wall, resulting in a well-stabilized cellular structure.

### 3.5. Mechanical Compressive Property

**Figure 15:**
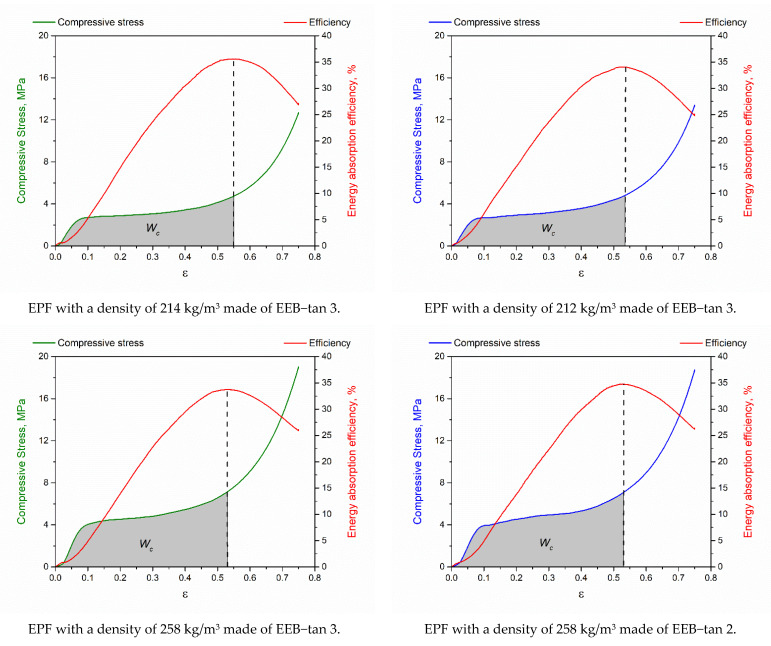
Compressive stress and energy absorption efficiency versus compressive strain of EPFs.

The compressive stress and strain curves are shown in Figure 15 and the specific mechanical compressive properties are reported in Table 5. Theses curves have three regions, 1st: initial linear–elastic; 2nd: a plateau with slightly increasing stress; and 3rd: a densification region. The mechanical compressive properties are comparable to those of epoxy composite foams produced by a non-continuous two-step foaming process [30], Rohacell WF and Airex R82 [26], and chopped-glass-fiber-reinforced polyisocyanurate foam [27]. Figure 15 shows the energy absorption efficiency and strain curves. The densification strain for EPFs ranged from 0.53 to 0.55 (53–55%). Interestingly, the effect of density was more pronounced for compressive modulus and strength but less visible for the densification strain. The difference in density from 210 kg/m^3^ to 258 kg/m^3^ may have been too small to affect the densification strain. Nevertheless, the strong fusion boundary in EPFs was also the main reason for the similar densification strain and the high energy absorption efficiency. 

The mechanical compressive properties are enhanced significantly with higher density. The differences in foam morphology and the effects of tan delta of EEB on the compressive strength are less obvious, which may indicate the stability in mechanical properties of the EPFs produced by both EEBs. The precuring time to achieve tan 3 and tan 2 varies from 64 to 87 min and can be considered a tolerance for the production of EEBs, which have almost similar mechanical compressive properties. This is a positive point for the possibility of scale-up.

### 3.6. Torsional Modulus and Glass Transition Temperature from DMA Test

**Figure 16:**
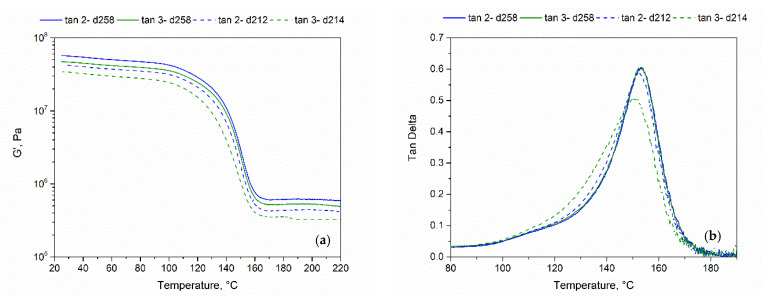
(**a**) Torsion storage modulus and (**b**) glass transition temperature of EPF measured from DMA.

The effect of EEB−tan 3 and EEB−tan 2 on cellular structure was evident with respect to the DMA torsional storage modulus, as shown in Figure 16a. EPFs made of EEB−tan 2 showed a higher torsional modulus than EPFs made of EEB−tan 3 at both densities. The EPF has a T_g_ in the range of 150–154 °C (Figure 16b), which is considered higher than most of the current commercial epoxy foams [29,30,31]. Sicomin provides a two-component resin for foaming epoxy with a T_g_ in the range of 60–142 °C [12]. Resoltech resin epoxy foams have a T_g_ in the range of 48–110 °C [13]. Thermoset polyurethane foam derived from lignin also has a typical T_g_ in the range of 38–117 °C [32].

## 4. Conclusions

A novel EEB was successfully produced by extrusion technique. The novel EEB is a carbamate-filled epoxy oligomer. A viscosity of 180 Pa·s is suitable for manual extrusion to form the extrudate into a round shape after further precuring. During precuring, DEN 431 reacts with Ancamine 2914UF to develop an oligomer with tan delta 3 and tan delta 2 at 60 °C. Both EEBs, with tan delta 3 and tan delta 2, respectively, were capable of foaming, expansion and fusion to produce high-strength EPF with a T_g_ of 150–154 °C and density of 212 kg/m^3^ and 258 kg/m^3^, respectively. An open-cell structure was obtained in both cases. The compressive modulus was in the range of 50–70 MPa. The specific strength at 50% compressive strain was 20–25 kNm/kg. To optimize the processing window for foaming, different foaming temperatures and EEBs with different tan deltas can be developed. The manufacturing technique of EEBs and the production of EPF investigated in this research demonstrates promise for scale-up. The first advantage is that EPF with different product shapes can be produced. The second advantage is that strong interbead fusion is achieved. A sandwich with high heat resistance and strength should be developed between this epoxy foam core and the epoxy face sheets, as they strongly adhere to each other. 

## Figures and Tables

**Table 1:** Compounding formula for 16.40 g of an epoxy mixture.

Code	Epoxy D.E.N 431	Ancamine 2914UF	IDPA.CO_2_	Theoretical CO_2_
EN.A.IDPA.CO_2_	12.20 g	1.00 g	3.20 g	4.1%

**Table 2:** Compounding formulae for 16.40 g of an epoxy mixture.

Code	Tan Delta	Target Density, kg/m^3^	Foaming Temperature, °C	Foaming Time, Minutes
Tan3-d210	3	210	160	60
Tan3-d258	3	258	160	60
Tan2-d210	2	210	160	60
Tan2-d258	2	258	160	60

**Table 3:** Rheological characteristics at foaming and gel point.

Rheological	G’_30 °C_, kPa	T_foaming_, °C	G’_foaming_, kPa	T_gel_ °C	G’_gel_, kPa
EEB−tan 3	30.1	127	0.3	130	0.33
EEB−tan 2	48.1	117	0.74	117	0.77

**Table 4:** Weight loss and the density of epoxy beads foaming at 140 °C, 150 °C and 160 °C.

Weight Loss %	f140 °C	f150 °C	f160 °C
EEB−tan 3	4.6 ± 0.2	4.7 ± 0.5	4.7 ± 0.4
EEB−tan 2	4.6 ± 0.2	4.5 ± 0.3	4.5 ± 0.2
**Density**	**f140 °C**	**f150 °C**	**f160 °C**
EEB−tan 3	167 ± 5	150 ± 12	130 ± 12
EEB−tan 2	169 ± 5	160 ± 5	132 ± 7

**Table 5:** Density, specific mechanical compressive properties, torsion storage modulus and T_g_ of EFB.

Sample	tan 3–d 214	tan 2–d 212	tan 3–d 258	tan 2–d 258
Weight loss, %	4.7 ± 0.1	4.8 ± 0.1	4.5 ± 0.1	4.6 ± 0.1
Density, kg·m^−3^	214 ± 1	211 ± 2	258 ± 1	258 ± 1
**Mechanical compressive properties**				
Compressive modulus, MPa	53.9 ± 4.7	51.8 ± 4.7	70.3 ± 2.3	65.3 ± 1.8
Specific compressive stress at 10%, MPa/kg·m^−3^	13.1 ± 0.4	12.6 ± 1.0	15.9 ± 0.5	15.6 ± 0.3
Specific compressive stress at 50%, MPa/kg·m^−3^	20.2 ± 1.2	20.8 ± 1.0	25.0 ± 0.9	24.3 ± 0.9
Energy consumption, *W_c_*, J/cm^3^	1.70 ± 0.10	1.71 ± 0.10	2.54 ± 0.03	2.51 ± 0.06
Densification strain, ε_d_, %	0.55 ± 0.01	0.54 ± 0.01	0.53 ± 0.01	0.53 ± 0.01
**Torsion dynamic mechanical properties**				
G’_@30°C_, MPa	33.9	42.0	46.6	56.4
T_g_ °C	150.6	152.6	153.0	153.9

## Data Availability

Not applicable.
